# The Week's Work

**Published:** 1911-04-15

**Authors:** 


### THE WEEK'S WORK.

### HOSPITALS OF TO-DAY.

With this number we begin a series of articles on Hospitals of To-day. The principle of selection is no arbitrary one, based upon the eminence or the obscurity, the amount of debt or the number of beds of these institutions, but an attempt has been made to single out those hospitals the position of which is peculiar either, as in the case of the Prince of Wales', Tottenham, because of the suddenness with which the hospital problem has developed, or, as will be seen in later articles, from the site, deed of foundation, or other cause. It so happens that the Prince of Wales', Tottenham, is considering the advisability of an appeal, but these articles are not necessarily of such a character. We have to thank those hospital secretaries who have already brought to our notice the position and needs of their institutions, which have enabled us to give in many cases not only the wants, but the character and personality which hospitals, like individuals, develop in the course of years.

### THE NEW SUPERINTENDENT AT EDINBURGH ROYAL INFIRMARY.

Lieut.-Colonel Sir Joseph Fayrer, Bart., R.A.M.C., has been appointed to fill the post of Superintendent of the Edinburgh Royal Infirmary made vacant by the retirement of Colonel Warburton. Sir Joseph Fayrer, who is fifty-two years of age, has had charge of the Military Hospital, Hong Kong, since 1909, and like his father, the first Baronet, has had a distinguished career in India in the Army Medical Corps. Sir Joseph is an M.D. of St. Andrews, besides being an F.R.C.S. of Edinburgh. He was educated at Rugby and Cambridge, and passed "first" into the Army in 1886, when he went at once to India. For three years he was in charge of civil hospitals at Muttra and was thanked by the Government for his services. In 1893 Sir Joseph was in Edinburgh again, and after serving with the Royal Horse Guards received the personal thanks of King Edward. In 1897 Sir Joseph Fayrer returned to India, and for over six years was in sole charge of the station hospitals at Sitapur and elsewhere. At the end of this period, further experience of English hospitals was gained by him as secretary to the Principal Medical Officer of the London district, after which the Duke of York's Military School, with five hundred boys, was placed under his care. It has long been the practice of the Edinburgh Royal Infirmary to have a military medical superintendent at the head of affairs, and though Sir Joseph possibly takes up the appointment rather late, there can be little doubt that he possesses qualities as distinguished as those of his predecessors. His experience has been gained in various countries, and it is as an Edinburgh man that he returns to the city. He will enter upon his duties on August 1, when he succeeds Colonel Warburton, who retires after twelve years of distinguished service, and who, like Sir Joseph, had gained much of his experience in India.

### HOSPITAL ARCHITECTURE.

The Corporation of Hamburg-Altona offers a prize to hospital architects, in open competition, for the best design for a new children's hospital which it is proposed to erect at Altona. The conditions stipulated are that the plans should be of such a nature as to admit of future extension when enlargement of the institution is found to be necessary. The scheme which the Corporation has in view is the building of a model children's hospital, with an attached but independent lying-in hospital. The available site is 500 cubic metres in extent, and the plans must comprise an administration block, a ward block or blocks, an infant ward block, a milk department, an out-patient department block, a laundry and kitchen block, and a mortuary block. Provision has to be made for seventy beds in the children's and fifty beds in the infants' wards. Details of the requirements and of the conditions of the competition—which is open to all hospital architects—are obtainable on application to Herr Direktor Feldmann, Katherinenstrasse, Altona. The new hospital, small as it is, offers scope for original treatment and an ingenious application of new principles of construction, and we look forward to seeing the prize designs.

### A NEW HOSPITAL FOR CONSTANTINOPLE.

The Turkish Government has decided to establish a large new hospital at Constantinople, a city which already possesses several interesting small institutions for the relief of the sick. The new building is to be a State institution, and the Evkaf Ministry has sent a special Commission to inspect the various new clinics which have been established during the last decade in America, in England, and on the Continent, with a view to deciding upon a model which would suit the special conditions of Constantinople. Kemal-Bey, who is the chief architectural expert attached to this mission, has expressed himself strongly in favour of the city hospital at Reinikendorf, near Berlin—one of the most interesting models in Germany—and the Commission has accordingly decided thoroughly to inspect this institution, and to arrange for the necessary equipment from German firms. The building of a large new hospital is always a matter of interest: when it is, however, planned to erect a North German model in a city like Constantinople, hospital constructors will follow with added interest the fortunes of the venture. Two of the newer Turkish hospitals are standing proofs of the fact that no hospital can be considered a success unless the architect allows for differences of climate and environment. It is to be hoped that those responsible for the construction of the projected institution will take care to profit by these examples.

### FLAPDOODLE.

The expressive American word which heads this paragraph has not yet obtained the rights of naturalisation in this country, but there are many terms which have been accepted as citizens long before the dictionary acknowledged them as such, and flapdoodle is now so generally used that no explanation of its meaning is necessary. At any rate, it is the word par excellence for the results of that *cacoethes scribendi* which impels so many members of the public to write to the press on hospital questions. We do not by any means deprecate the discussion of hospitals or medical questions in the lay press. We have never taken up the hierarchical position that such subjects are sacrosanct, only to be touched by those who are expert: on the contrary we have repeatedly stated that what is urgently wanted in this country is a more active, a more intelligent interest in the condition of hospitals and in the work that is done in them. But ill-informed, intemperate, and opiniated statements do more harm than good, and if the intelligent interest of the public is to be judged from the correspondence on certain hospital subjects that has recently been printed in a provincial contemporary, we fear that those who deny the right of the public to discuss such questions have a reasonable ground for their desire to keep such matters limited to experts. In one letter we find the statement that the public imagines that there ought to be some check—and a great check—to stop the increasing death-rates in hospitals; in another an argument that the washing of patients is a dangerous proceeding which is liable to result in "possible developments." Three parts of this correspondence is flapdoodle; the fourth part is ignorance parading itself as criticism.

### AND ITS CURE.

One or two things it is imperative that all hospital workers should grasp. The public needs education in facts with regard to hospitals. Nor is it always the large, the tremendous fact that tells; the great things, as Emerson said, are the near ones. One of the facts which the public, in spite of the mass of writing that has been devoted to a consideration of the subject, has not yet grasped, is that a patient's chances to escape death when he is stricken with a serious illness are ten times better when he goes to a modern hospital than if he stays at home. Another equally important fact is that the chances of such a patient, especially a surgical patient, are, so far as the hospital is concerned, bettering every year. The death-rate in hospitals ten years ago was much larger than it is to-day; it will no doubt be much less in 1920 than it is now. The points in a discussion on comparative values are just points which the layman is apt to overlook. Curability, necessity, and emergency are entirely different things, but a hospital cannot, and ought not to, afford to differentiate between them in making up its general statistics, unless every hospital in the kingdom does the same. Therefore there remains a risk that the layman will continue to think ill of a hospital that returns five deaths in one hundred cases of operation for appendicitis, especially when his own medical man assures him that the death-rate in that operation is one in five hundred. To the hospital workers the fallacy is obvious: the one figure includes *all* operations—the fearful general peritonitis case together with the easy, harmless removal of the appendix à froid; the other is merely the statistics of successive interval cases which do not fairly put the position at all. These differences should be explained to the public. If the profession will not do it, the hospital workers must attempt the task. We see a great future before hospital secretaries as interpreters between the medical profession and the public.

### EMINENT CHAIRMEN SERIES.

The next article of this series will deal with Mr. Perceval Nairne, Chairman of the Dreadnought Hospital, Greenwich, and with the work of that institution. Owing to the Easter holidays we are compelled to postpone the article, which was to have appeared in the present issue.

